# Leaves, not roots or floral tissue, are the main site of rapid, external pressure-induced ABA biosynthesis in angiosperms

**DOI:** 10.1093/jxb/erx480

**Published:** 2018-01-29

**Authors:** Feng-Ping Zhang, Frances Sussmilch, David S Nichols, Amanda A Cardoso, Timothy J Brodribb, Scott A M McAdam

**Affiliations:** 1 Key Laboratory of Economic Plants and Biotechnology, Kunming Institute of Botany, Chinese Academy of Sciences, China; 2 Institute for Molecular Plant Physiology and Biophysics, University of Würzburg, Germany; 3 School of Biological Sciences, University of Tasmania, Australia; 4 Central Science Laboratory, University of Tasmania, Australia; 5 Departamento de Biologia Vegetal, Universidade Federal de Viçosa, Campus Universitário, Brasil; 6 Department of Botany and Plant Pathology and Purdue Center for Plant Biology, Purdue University, USA

**Keywords:** Abscisic acid, cell volume, 9’-*cis*-epoxycarotenoid dioxygenase, flowers, roots, stomata

## Abstract

Rapid biosynthesis of abscisic acid (ABA) in the leaf, triggered by a decrease in cell volume, is essential for a functional stomatal response. However, it is not known whether rapid biosynthesis of ABA is also triggered in other plant tissues. Through the application of external pressure to flower, root, and leaf tissues, we test whether a reduction in cell volume can trigger rapid increases in ABA levels across the plant body in two species, *Solanum lycopersicum* and *Passiflora tarminiana*. Our results show that, in contrast to rapid ABA synthesis in the leaf, flower and root tissue did not show a significant, increase in ABA level in response to a drop in cell volume over a short time frame, suggesting that rapid ABA biosynthesis occurs only in leaf, not in flower or root tissues. A gene encoding the key, rate-limiting carotenoid cleavage enzyme (9-*cis*-epoxycarotenoid dioxygenase, NCED) in the ABA biosynthetic pathway in *S*. *lycopersicum*, *NCED1*, was upregulated to a lesser degree in flowers and roots compared with leaves in response to applied pressure. In both species, floral tissues contained substantially lower levels of the NCED substrate 9’-*cis*-neoxanthin than leaves, and this ABA precursor could not be detected in roots. Slow and minimal ABA biosynthesis was detected after 2 h in petals, indicating that floral tissue is capable of synthesizing ABA in response to sustained water deficit. Our results indicate that rapid ABA biosynthesis predominantly occurs in the leaves, and not in other tissues.

## Introduction

The plant hormone abscisic acid (ABA) plays a critical role during many phases of the plant life cycle, regulating responses to various environmental signals as well as endogenous cues including water limitation (Wright and Hiron, 1969; Pierce and Raschke, 1980; Henson 1982; McAdam and Brodribb, 2015), seed development and dormancy (Zeevaart and Creelman, 1988), and sex determination (Hickok *et al.*, 1995; McAdam *et al.*, 2016*a*). The processes regulated by ABA signaling in flowering plants are highly distinct in terms of target organ and functional role, including the regulation of leaf transpiration (Li *et al.*, 2000). In the leaves of seed plants, ABA regulates transpiration by activating anion channels in the guard cells that flank the stomatal pore, causing cell turgor loss and stomatal closure (Schroeder *et al.*, 2001; Geiger *et al.*, 2009). In angiosperms, this regulation of stomatal aperture by ABA is an essential means by which these plants regulate diurnal gas exchange, with changes in ABA levels driving stomatal responses to leaf-to-air vapor pressure deficit (VPD) (Bauerle *et al.*, 2004; McAdam and Brodribb, 2015; Buckley, 2016; Qiu *et al.*, 2017). In order to regulate stomatal response during changes in VPD, angiosperms have evolved a mechanism for rapid ABA biosynthesis (Bauerle *et al.*, 2004; McAdam and Brodribb, 2015; McAdam *et al.*, 2016*b*).

Recently, studies have shown that foliar ABA biosynthesis is actively up-regulated in response to a drop in cell volume, similar to what occurs when a leaf is exposed to an increase in VPD, rapidly triggering stomatal closure (McAdam and Brodribb, 2016; McAdam *et al.*, 2016*b*; Sack *et al.*, 2017; Sussmilch *et al.*, 2017). ABA in plants is produced via oxidative cleavage of epoxycarotenoids. The 9-*cis*-epoxycarotenoid dioxygenase (NCED) enzyme cleaves C40 9-*cis*-epoxycarotenoids stored within plastids to release xanthoxin, a C15 intermediate. This step is the rate-limiting step in the ABA biosynthetic pathway and, at high VPD, or when leaves are briefly exposed to a drop in leaf turgor, there is a rapid, significant increase in the expression of at least one key *NCED* gene encoding this enzyme (Bauer *et al.*, 2013*a*; Pantin *et al.*, 2013; Sussmilch *et al.*, 2017).

While the essential role of ABA in regulating diurnal stomatal aperture in angiosperms requires a rapid means of up-regulating ABA biosynthesis, it remains unclear whether ABA can be synthesized in other tissues at similar speeds. Two key organs that require investigation are the flowers, the survival of which is essential for plant reproduction, and the roots, which have been at the center of a long-running and extensive debate about the biosynthetic origin of ABA particularly for stomatal regulation (Zhang *et al.*, 1987; Holbrook *et al.*, 2002; McAdam *et al.*, 2016*c*).

Flowers are relatively short-lived, and they do not assimilate substantial amounts of carbon, lessening the need for atmospheric gas exchange and thus high rates of transpiration (Roddy and Dawson, 2012; Teixido and Valladares, 2014). Because flowers often face desiccating conditions that would lead to wilting, they must maintain a positive balance between water supply and loss, as well as high turgor throughout flowing. In addition, it appears that flowers may be more hydraulically vulnerable than leaves, which places even more selective pressure on flowers to maintain minimal water loss rates compared with leaves (Zhang and Brodribb, 2017). Flowers are developmentally independent from leaves, and they also appear to follow different evolutionary trajectories in water transport and vein density (Roddy *et al.*, 2013). Thus, flowers and leaves may experience different turgor pressures and water balances, and, given the limited gas exchange in flowers compared with leaves, there may be no selective pressure to maintain a rapid cell volume-triggered ABA synthesis pathway in floral tissues.

Early experiments on ABA biosynthesis using bench-dried whole plants revealed an accumulation of ABA in both the leaves and root tips (Zhang *et al.*, 1987). This observation of ABA accumulation in roots tips, combined with later studies showing stomatal closure in split-root drought experiments that apparently could not be explained by reduced leaf water status (Davies and Zhang, 1991), led to the root-sourced ABA theory. This theory gained widespread acceptance (Tardieu and Davies 1993) until reciprocal grafting studies with ABA biosynthetic mutants revealed that stomatal aperture is predominantly regulated by leaf-sourced ABA (Holbrook *et al.*, 2002; Christmann *et al.*, 2007; McAdam *et al.*, 2016*b*). Subsequently a number of studies have demonstrated that not only are leaves the primary source of ABA biosynthesis during water limitation, but roots lack the endogenous carotenoid precursors required for substantial ABA biosynthesis during drought (Manzi *et al.*, 2016). While the root-sourced ABA theory cannot explain ABA accumulation in leaves during water limitation, localized synthesis in roots may occur to a small degree (Manzi *et al.*, 2016). Whether this localized synthesis is triggered by a reduction in cell volume similar to the leaf remains to be tested.

In this study, we investigated whether rapid accumulation of ABA is triggered by a reduction in cell volume across all plant tissues by the application of external pressure in two angiosperm species, *Solanum lycopersicum* and *Passiflora tarminiana*. We specifically tested whether flower and root cell volume provides a quantitative regulatory signal for ABA biosynthesis over a short time frame as in leaves in *S*. *lycopersicum* and *P*. *tarminiana*. We used precise physicochemical quantification of ABA levels to detect changes in flower, root, and leaf ABA when the volume of cells from the respective tissues was reduced for a short period of time, by the application of external pressure. In addition, we measured changes in the expression of the rate-limiting ABA biosynthetic gene, *NCED1*, in leaves, flowers, and roots in *S*. *lycopersicum* following this change in cell volume.

## Materials and methods

### Plant material

Two angiosperm species, *S. lycopersicum* and *P. tarminiana*, were selected for this experiment. *Solanum lycopersicum* was chosen in particular because we could monitor the expression of the key gene responsible for catalyzing the rate-limiting step in the ABA biosynthetic pathway, in this case *NCED1* (characterized by the classical ABA biosynthetic mutant, *notabilis*) (Burbidge *et al.*, 1999). All experiments were conducted between 11.00 h and 14.00 h. Individual *S*. *lycopersicum* plants were grown in 14 cm slim-line pots in potting mix, in controlled-environment growth cabinets. Conditions in the growth cabinet were regulated at 25 °C/16 °C day/night temperature with a 16 h photoperiod, provided by mixed incandescent and fluorescent lights, ensuring a minimum of 300 μmol quanta m^–2^ s^–1^ at the pot surface. All plants were watered daily and received weekly applications of liquid fertilizer (Aquasol, Hortico Ltd, Padstow, NSW, Australia). *Passiflora tarminiana* was grown outside in the gardens of the University of Tasmania.

### Stomatal quantification

Leaves and flowers were collected simultaneously and transported to the laboratory for microscopic analysis. Approximately 10 mm^2^ sections were taken from midway between the leaf midrib and margin. We collected multiple 1 cm^2^ sections from all parts of bracts, petals, and sepals. For structures of sepals and petals of *S*. *lycopersicum* that were smaller, we sampled the entire petal and sepal. Sections were then bleached in commercial household bleach (50 g l^–1^ sodium hypochlorite and 13 g l^–1^ sodium hydroxide) until clear. Bleach was removed by washing, and sections were stained in 1% toluidine blue for 30 s to color the lignin-rich veins. Sections were mounted in phenol glycerine jelly and photographed with a Nikon Digital Sight DS-L1 camera (Melville, NY, USA) mounted on a Leica DM 1000 microscope (Nussloch, Germany). Stomatal density was measured from these paradermal sections.

### Assessing ABA synthetic capacity across tissues

Branches or compound leaves, flowers, and roots were excised from fully hydrated plants that had not been exposed to a VPD higher than 1.0 kPa for at least 24 h. Samples of tissue adjacent to the manipulated tissue were taken for ABA quantification to act as a control. Tissue was then wrapped in a damp paper towel to prevent dehydration by transpiration and enclosed in a Scholander pressure chamber with the excised end emerging from the chamber. A positive pressure provided by compressed air was applied to the samples to a prescribed MPa (0.5, 1.5, 2.0, and 2.5 MPa for both *P. tarminiana* and *S. lycopersicum*; in addition to 1.0 MPa for *S. lycopersicum* only) for 30 min. For *S. lycopersicum*, a positive pressure provided by compressed air was applied to leaf, flower, and root samples to 0.5, 1.0, 1.5 2.0, and 2.5 MPa for 30 min. Increases and decreases in pressure were made gradually (<0.1 bar s^–1^). Control samples were also included where tissue was enclosed in the chamber without pressurization. Following pressurization, a sample was again taken for ABA quantification. A microscope was used to monitor the excised end of the leaf, flower, or root during the experiment to ensure that tissue in the chamber did not desiccate to a water potential below the pressure applied.

### ABA and carotenoid extraction, purification, and quantification

ABA was extracted, purified, and physicochemically quantified by the high precision method of ultra-performance LC–tandem MS with an added internal standard according to the method of McAdam (2015). The levels of the carotenoid ABA precursors (including 9'-*cis*-neoxanthin and 9-*cis*-violaxanthin) were extracted from similar tissue harvested for ABA analysis but covered instead in 1–2 ml of cold (–20 °C) acetone. Tissue was then homogenized as per samples for ABA analysis and allowed to extract overnight, in the dark, at 4 °C. Samples were then centrifuged at 10000 *g*, after which the supernatant was removed and the volume was measured. Sufficient water was then added so that carotenoids were suspended in an 80% acetone solution in water (v/v). Carotenoid levels were analyzed using a Waters Acquity H-Class UPLC instrument coupled to a Waters photodiode array (PDA) Detector. A Waters Acquity UPLC beh C_18_ column (2.1 mm×100 mm×1.7 μm) was used. The mobile phase consisted of three solvents: water (solvent A), acetonitrile (solvent B), and methanol:hexane (80:20, v/v; solvent C). The UPLC program was initially 19%A: 81%B: 0%C which was held for 3.2 min before a gradient to 0%A: 50%B: 50%C at 10 min, and this was followed by immediate re-equilibration to starting conditions for 3 min. The flow rate was 0.45 ml min^−1^, the column was held at 35 °C, and the sample compartment was at 6 °C.

The PDA detector acquired three-dimensional data over the wavelength range 230–500 nm with a resolution of 1.2 nm at five points per second. Single wavelength channels were also acquired at 497, 440, and 440 nm background corrected under similar conditions. Data were analyzed and processed using MassLynx software. The identification of 9'-*cis*-neoxanthin and 9-*cis*-violaxanthin was achieved by comparison of the experimentally obtained UV spectrum (230–500 nm) with the published literature and a freshly obtained plant leaf acetone extract. Likewise, the purity of the chromatographically resolved peaks in samples was assessed by the reproducibility of the experimentally obtained UV spectrum (230–500 nm). Levels of 9'-*cis*-neoxanthin were quantified by comparing the peak areas of this compound with the peak area of a standard of 400 ng of β-carotene. A relative response factor for the difference in molar extinction coefficients between 9'-*cis*-neoxanthin and 9-*cis*-violaxanthin with β-carotene was calculated from the literature values of molar extinction coefficients for each compound. Beer’s Law was then applied to calculate the concentration of 9'-*cis*-neoxanthin and 9-*cis*-violaxanthin, based on the response of the β-carotene standard solution (Goericke and Repta, 1993). No 9-*cis*-violaxanthin was detected in any tissues.

### Gene expression analysis

Quantitative reverse transcription–PCR analysis was used to determine the expression of *NCED1* in *S*. *lycopersicum,* in tissue that was exposed to 1.5 MPa for 30 min relative to controls. After exposure to external pressure, tissue was snap-frozen in liquid nitrogen and stored at –70 °C until further processing. RNA was extracted using the Agilent Plant RNA Isolation Mini Kit (Agilent Technologies Inc., Wilmington, DE, USA), and RNA concentrations were determined using a NanoDrop 8000 (Thermo Scientific, Waltham, MA, USA). Reverse transcription was conducted in 20 μl with 1 μg of total RNA using the Tetro cDNA synthesis kit (Bioline, London, UK) according to the manufacturer’s instructions. Reverse transcriptase-negative (no enzyme) controls were performed to monitor for contamination with genomic DNA. First-strand cDNA was diluted five times, and 2 μl was used in each PCR. Reactions using SYBR green chemistry (SensiFAST, Bioline) were set up with a CAS-1200N robotic liquid handling system (Corbett Research, Mortlake, NSW, Australia) and run for 50 cycles in a Rotor-Gene Q (Qiagen, Valencia, CA, USA). Three to four biological replicates and two technical replicates were performed for each sample. Transcript levels for the *NCED1* gene were evaluated against a previously identified housekeeping gene that encodes a TIP41-like family protein (SGN-U584254/Siktc10g049850.1.1) (Dekkers *et al.*, 2012) with primers CATGCCTAGTGGTTGGTTCC and AGACAAGGCCTGAAATGTGG. This *TIP41*-like gene was found to be stably expressed. Primer details for *NCED1* are as described in McAdam *et al.* (2016*b*).

## Results

**Fig. 1.**
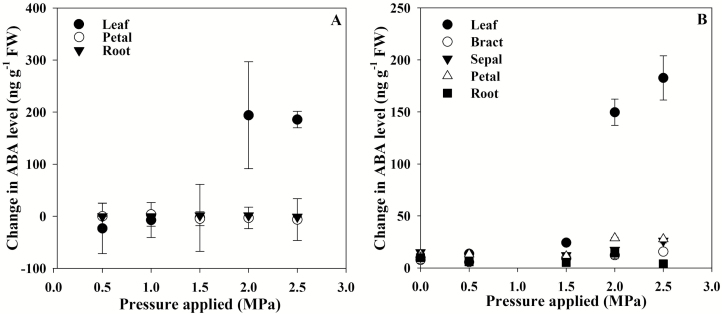
The mean change in ABA level (*n*=3, ±SE) for leaves, roots, and flowers, including floral parts, of *Solanum lycopersicum* (A) and *Passiflora tarminiana* (B) after 30 min of applied external pressure.

When the cell volume of leaves, flowers, and roots of *S. lycopersicum* and *P. tarminiana* was lowered after short-term exposure to external pressure, only leaves showed a substantial increase in ABA levels (Fig. 1). In both species, a clear threshold trigger point for ABA biosynthesis was apparent (Fig. 1). In *S*. *lycopersicum*, 2.0 MPa of external pressure was required to trigger foliar ABA biosynthesis in 30 min, with ABA levels in leaves exposed to this external pressure increasing by >200 ng g^–1^ FW (Fig. 1A). Similarly, in *P*. *tarminiana*, 2.0 MPa of external pressure was required to trigger foliar ABA biosynthesis, with levels increasing by ~150 ng g^–1^ FW (Fig. 1B). In *S. lycopersicum* flowers and roots, and in *P. tarminiana* roots, ABA levels did not increase significantly within this time frame, following the application of external pressure and the reduction in turgor (Fig. 1). In *P. tarminiana* flowers, ABA levels in all tissues, including bracts, sepals, and petals, increased slightly (by <30 ng g^-1^ FW) at the same trigger point as found in leaves, namely 2.0 MPa (Fig. 1B).

**Fig. 2.**
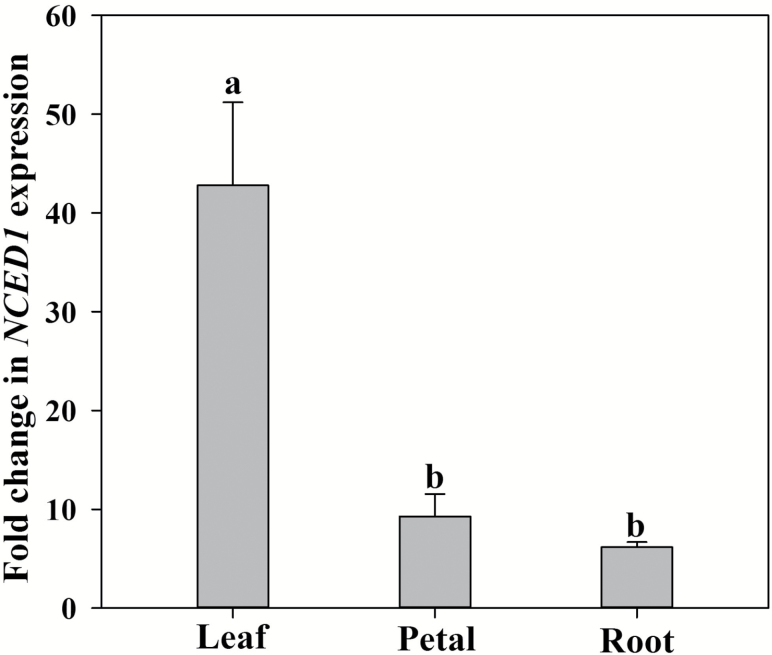
The fold change in relative expression of the *NCED1* gene in leaf, petal, and root of *Solanum lycopersicum* after 30 min of applied pressure (*n*=4, ±SE). Different letters denote significant differences between means.

The application of 2.0 MPa external pressure to leaves, petals, and roots trigged a significant upregulation of the expression of the *NCED1* gene in each of these tissues in *S. lycopersicum* (Fig. 2). However, major differences in the degree of transcriptional upregulation of this gene were evident between tissues. In the leaves, *NCED1* expression increased >40-fold, whereas in the petals and roots, *NCED1* expression increased <10-fold after 30 min of applied external pressure and reduction in cell turgor (Fig. 2).

**Fig. 3.**
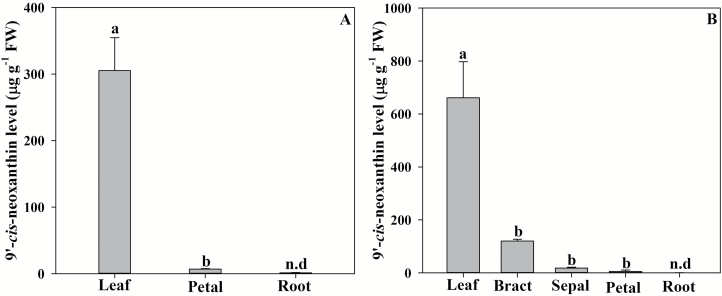
The mean levels of the carotenoid 9'-*cis*-neoxanthin (*n*=3 ±SE) in leaves, roots, and flowers, including floral parts, of *Solanum lycopersicum* (A) and *Passiflora tarminiana* (B). Different letters denote significant differences between means. n.d., not detectable.

Major differences between tissues in the pool of the key ABA biosynthetic carotenoid precursor 9'-*cis*-neoxanthin were found between tissues in both *S*. *lycopersicum* and *P. tarminiana* (Fig. 3). In both species, leaves contained by far the highest levels of 9'-*cis*-neoxanthin, with *S. lycopersicum* leaves containing ~320 µg g^–1^ FW and *P*. *tarminiana* leaves containing ~620 µg g^–1^ FW (Fig. 3). In both species, floral tissues contained substantially less 9'-*cis*-neoxanthin, with very low levels (only 6.88 µg g^–1^ FW) detected in *S. lycopersicum* petals (Fig. 3A). In *P. tarminiana*, 9'-*cis*-neoxanthin levels decreased through the orders of floral tissues, with bracts containing around a fifth of the foliar level of 9'-*cis*-neoxanthin, declining to 5.08 µg g^–1^ FW in petals (Fig. 3B). In both species, 9'-*cis*-neoxanthin was not detectable in roots (Fig. 3).The alternative precursor 9-*cis*-violaxanthin was not detectable in any tissues.

**Fig. 4.**
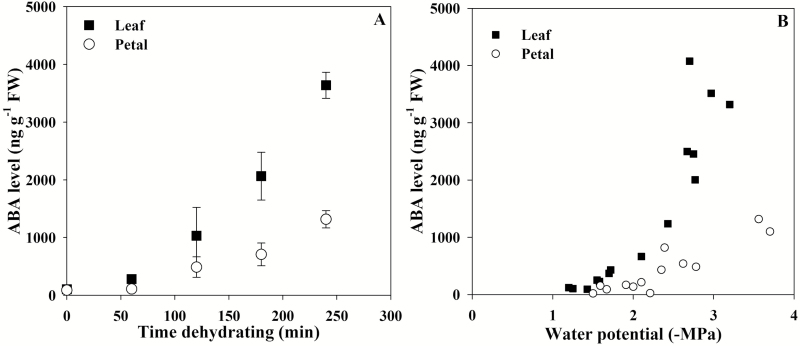
Evidence of limited and slow ABA biosynthesis in the flowers of *Passiflora tarminiana* (*n*=3 ±SE in A and *n*=3 in B).

While rapid ABA biosynthesis following a reduction in petal cell volume was not detected in either species (Fig. 1), slow ABA biosynthesis over many hours did occur in the petals of *P. tarminiana* (Fig. 4). After drying excised flowers and leaves on the bench for at least 3 h, ABA levels were found to increase after 2 h and to a lesser level in petals compared with leaves (Fig. 4A), despite an apparently similar leaf water potential trigger for ABA biosynthesis (Fig. 4B).

## Discussion

### Leaves are a primary source of ABA following short-term reductions in turgor

In leaves it is widely accepted that ABA levels increase during water stress, playing a major role in closing stomata as leaves lose turgor (Wright and Hiron, 1969; Pierce and Raschke, 1980; Henson, 1982; McAdam and Brodribb, 2015). Here, using a recently described technique of modifying cell volume by applying external pressure (McAdam and Brodribb, 2016; Sack *et al.*, 2017), we found that leaves display the most substantial increases in ABA levels in response to a reduction in cell volume over a 30 min time frame. The substantial increases in foliar ABA level following a reduction in leaf cell volume occurred at the same time as a corresponding increase in the expression of a key gene encoding the rate-limiting enzyme in the ABA biosynthetic pathway, *NCED1*, in *S. lycopersicum* in these tissues. As expression of this gene is a major determinant of ABA levels in *S. lycopersicum* (Burbidge *et al.*, 1999), these results indicate that the increases in ABA levels observed here are due to increased ABA biosynthesis as opposed to release of ABA from fettered stored pools (Lee *et al.*, 2006). This conclusion is also supported by the previous finding that the stores of ABA-glucose ester increase, rather than decrease, under rapid VPD transitions, and increased ABA biosynthesis, facilitated by increased *NCED1* expression, instead accounts for the rapid increase in ABA level under these conditions (McAdam *et al.*, 2016*b*). In contrast to leaves, flowers displayed either no rapid increase in ABA levels in *S*. *lycopersicum* or extremely minimal increases in ABA level in *P. tarminiana* after a 30 min reduction in cell volume (Fig. 1). Roots did not show an increase in ABA levels after this reduction in cell volume. These results raise the possibility that there may be tissue-specific differences in activity of the long hypothesized, but as yet uncharacterized, sensor that activates ABA biosynthesis (Pierce and Raschke, 1980; Creelman and Mullet, 1991; Sussmilch *et al.*, 2017), recently shown to detect a decrease in cellular volume (Sack *et al.*, 2017). In addition to tissue specificity, there may also be considerable differences across species in the sensitivity of the cell volume sensor for ABA biosynthesis (McAdam and Brodribb, 2016); whether this difference drives iso- or anisohydric variation in stomatal responses to water deficit remains to be tested.

### All tissues have a cell volume sensor that can trigger the expression of a key gene encoding the rate-limiting ABA biosynthetic enzyme

Despite major differences in the synthesis of ABA across tissues, an analysis of the expression profile of a key, rate-limiting gene in the ABA biosynthetic pathway, *NCED1*, in *S. lycopersicum* revealed significant increases in expression in all tissues after 30 min of reduced cell volume. This result suggests that all tissues have a cell volume-sensing pathway including a transcriptional regulator that is essential for regulating ABA levels. However, compared with petals and roots, leaves displayed a significantly higher level of transcriptional induction of *NCED1* after 30 min, which does suggest some degree of tissue specificity in this pathway.

### Differences in the spatial pattern of ABA level accumulation mirror the spatial pattern of carotenoid precursors

An increase in *NCED* expression in roots during drought has been observed in a number of species including citrus (Manzi *et al.*, 2016). In this study, an increase in the expression of *NCED1* in tissues other than leaves of *S. lycopersicum* did not result in a rapid increase in ABA levels. In citrus, Manzi *et al.* (2016) recently demonstrated that roots lack sufficient levels of key carotenoid precursors for substantial localized ABA biosynthesis. In agreement with these recent studies, we found major differences in the pools of 9'-*cis*-neoxanthin between tissues, with extremely low levels found in the roots. This limited pool of 9'-*cis*-neoxanthin in flowers and especially roots mirrored a lack of substantial ABA biosynthesis in these tissues after a short-term reduction in cell volume in both species. Our results further add to a growing body of evidence that has suggested that ABA is not substantially synthesized in roots (Kudoyarova *et al.*, 2011), challenging a long-held dogma that roots are the primary source for the synthesis of ABA (Zhang *et al.* 1987).

Unlike *S. lycopersicum*, ABA levels did accumulate over both a short and longer period of time in the flowers of *P. tarminiana*, albeit to a lesser extent than in leaves. This probably reflects reduced but not absent levels of 9'-*cis*-neoxanthin in these tissues. Whether or not this minimal, yet clear, floral accumulation of ABA is functionally relevant for stomatal control in this species remains to be tested; however, it should be noted that *P*. *tarminiana* does possess a very low density of stomata in all floral organs (see Supplementary Fig. S1 at *JXB* online). It is unlikely that the reduced levels of ABA in the flowers of this species were a direct result of a reduced number of stomata (although guard cells have been suggested to be a main site of ABA biosynthesis in the plant; Bauer *et al.*, 2013*b*), because *S. lycopersicum* also has stomata on petals (Supplementary Fig. S1) yet does not show a floral increase in ABA levels.

### Conclusion

We find that ABA levels increase most substantially in leaves following a short-term reduction in cell volume generated by the application of external pressure. This major increase in ABA levels in leaves compared with other tissues including flowers and roots is likely to be the result of both increased levels of ABA carotenoid precursor and enhanced upregulation of expression of a gene encoding the rate-limiting enzyme in the ABA biosynthetic pathway. We find that all tissues possess the capacity to upregulate the expression of this critical gene, but that pools of ABA precursors vary dramatically across tissues, mirroring the differences in the degree of rapid ABA synthesis.

## Supplementary data

Supplementary data are available at *JXB* online.

Fig. S1. The stomatal density of flower and leaf of *Solanum lycopersicum* (A) and *Passiflora tarminiana* (B).

Supplementary Figure S1
