# Supplementary material for: Leaves, not roots or floral tissue, are the main site of rapid, external pressure-induced ABA biosynthesis in angiosperms
Source: J Exp Bot. 2018 Jan 29;69(5):1261–7. doi: 10.1093/jxb/erx480 (PMC6018962; doi:10.1093/jxb/erx480)
Supplement: Supplementary Figure S1 [file erx480_suppl_supplementary_figure_s1.pdf]

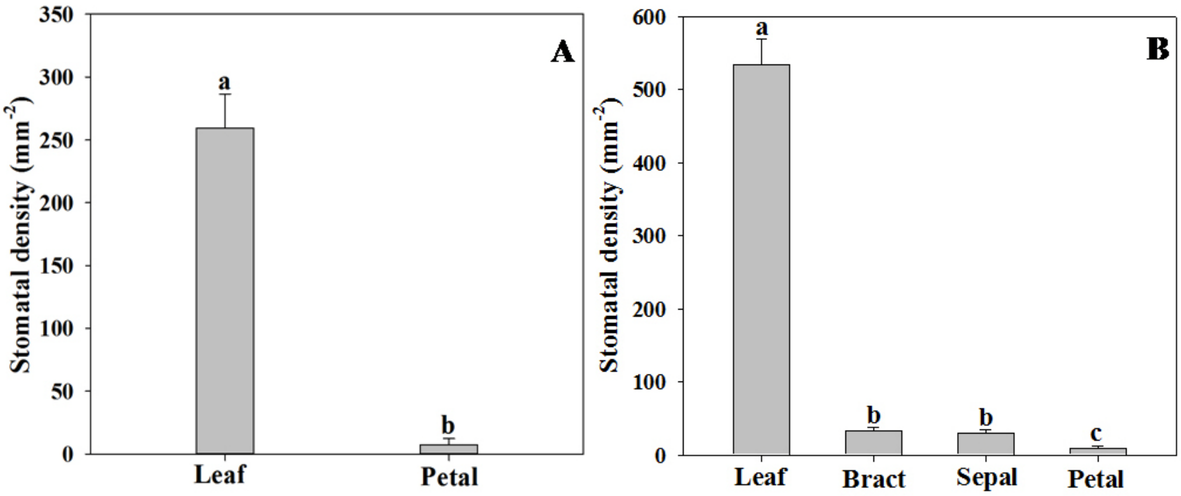

Supplementary Fig. S1

The stomatal density of flower and leaf of *Solanum lycopersicum* (A) and *Passiflora tarminiana* (B). Different letters denote significant differences between means.
